# The Differential Expression of Cide Family Members is Associated with Nafld Progression from Steatosis to Steatohepatitis

**DOI:** 10.1038/s41598-019-43928-7

**Published:** 2019-05-16

**Authors:** Arnaud Sans, Stéphanie Bonnafous, Déborah Rousseau, Stéphanie Patouraux, Clémence M. Canivet, Pierre S. Leclere, Jeanne Tran-Van-Nhieu, Carmelo Luci, Béatrice Bailly-Maitre, Xu Xu, Ann-Hwee Lee, Kaori Minehira, Rodolphe Anty, Albert Tran, Antonio Iannelli, Philippe Gual

**Affiliations:** 1 Université Côte d’Azur, INSERM, U1065, C3M, Nice, France; 2 Université Côte d’Azur, CHU, INSERM, U1065, C3M, Nice, France; 3 HU Henri Mondor, Department of Pathology, AP-HP - Université Paris Est Créteil, Créteil, France; 4 Weill Cornell Medicine, Department of Medicine, Division of Gastroenterology and Hepatology, New York, USA; 5 Department of Pathology and Laboratory Medicine, Weill Cornell Medical College, New York, USA; 6 University of Lausanne, Department of Physiology, Lausanne, Switzerland

**Keywords:** Non-alcoholic fatty liver disease, Non-alcoholic steatohepatitis

## Abstract

Improved understanding of the molecular mechanisms responsible for the progression from a “non-pathogenic” steatotic state to Non-Alcoholic Steatohepatitis is an important clinical requirement. The cell death-inducing DFF45 like effector (CIDE) family members (A, B and FSP27) regulate hepatic lipid homeostasis by controlling lipid droplet growth and/or VLDL production. However, CIDE proteins, particularly FSP27, have a dual role in that they also regulate cell death. We here report that the hepatic expression of CIDEA and FSP27 (α/β) was similarly upregulated in a dietary mouse model of obesity-mediated hepatic steatosis. In contrast, CIDEA expression decreased, but FSP27-β expression strongly increased in a dietary mouse model of steatohepatitis. The inverse expression pattern of CIDEA and FSP27β was amplified with the increasing severity of the liver inflammation and injury. In obese patients, the hepatic CIDEC2 (human homologue of mouse FSP27β) expression strongly correlated with the NAFLD activity score and liver injury. The hepatic expression of CIDEA tended to increase with obesity, but decreased with NAFLD severity. In hepatic cell lines, the downregulation of FSP27β resulted in the fractionation of lipid droplets, whereas its overexpression decreased the expression of the anti-apoptotic BCL2 marker. This, in turn, sensitized cells to apoptosis in response to TNF α and saturated fatty acid. Considered together, our animal, human and *in vitro* studies indicate that differential expression of FSP27β/CIDEC2 and CIDEA is related to NAFLD progression and liver injury.

## Introduction

Non Alcoholic Fatty Liver Diseases (NAFLD) is a major public health concern with global prevalence ranging from 22% to 28%^1^. NAFLD is increasingly recognized as the most common chronic liver disease^1^. The spectrum of the hepatic diseases ranges from steatosis (fatty liver) to nonalcoholic steatohepatitis (NASH) (steatosis, inflammation, liver injury) and subsequently to the activation of fibrogenic pathways, which correlates with a high risk of developing cirrhosis and hepatocellular carcinoma. Despite lifestyle changes and bariatric surgery for severe/morbid obesity, the treatment of NAFLD (NASH) is still limited because of the lack of effective pharmacological treatment as well as lack of effective and practical diagnostic tools. NAFLD is associated with obesity and metabolic syndrome and the presence of type 2 diabetes mellitus can increase the risk of liver diseases^2,3^. Inversely, NAFLD is also a risk factor for many metabolic diseases, including type 2 diabetes^4^ and cardiovascular disease^5^.

The mechanisms underlying the transition from steatosis to NASH are multifactorial and not fully elucidated. The hepatocyte accumulation of triglycerides in lipid droplets is a protective mechanism that buffers free fatty acids and prevents lipotoxicity^6^. However, this protective mechanism can be overwhelmed. To illustrate this, it has been reported that the inhibition of the triglyceride synthesis via the targeting of diacylglycerol acyltransferase 2 (DGAT2) improves hepatic steatosis but exacerbates liver damage and fibrosis in obese mice with NASH^7^. The vulnerable fatty hepatocytes generate danger signals including the release of alarmins and damage-associated molecular patterns as well as the enrichment of apoptotic bodies. This activation of sterile inflammation is involved in the initiation of a vicious cycle, where inflammation enhances hepatocyte death and *vice-versa*.

The cell death-inducing DFF45 like effector (CIDE) protein family, including CIDEA, CIDEB and CIDEC/fat-specific protein 27 (FSP27), are lipid droplet-associated proteins. Since these proteins regulate lipid droplet synthesis and hepatic lipid homeostasis, they could be key players in the control of NAFLD progression^8^. The CIDEA is a short-lived protein that is mainly controlled by the sterol regulatory element binding protein-1c (SREBP-1c; master transcription factor regulated *de novo* lipogenesis) and shows increased expression in the case of hepatic steatosis^9,10^. Its overexpression in mouse liver resulted in augmented hepatic lipid accumulation and the formation of large lipid droplets. In contrast, the hepatic knockdown of CIDEA in obese mice resulted in significantly reduced hepatic lipid accumulation and smaller lipid droplets^10^. CIDEB is highly and constitutively expressed in the liver and controls both insulin sensitivity and VLDL maturation^11,12^. CIDEC, also known as fat-specific protein 27 (FSP27) in mouse, is also highly expressed in fatty liver and knock-down of its expression in fatty liver tissue ameliorated hepatic steatosis^13–17^. Its overexpression in hepatic cells enhanced the triglyceride content of lipid droplets^17^. The two FSP27α and β isoforms of mouse FSP27 (corresponding to human CIDEC1 and 2, respectively) are regulated by the peroxisome proliferator-activated receptor gamma (PPARγ)^14–17^ and Cyclic-AMP-responsive-element-binding protein H (CREBH)^18,19^. The CIDE protein family can also mediate cell death depending on their expression levels and cellular localization^20–23^.

The relative expression levels of the three CIDE members over the transition period from simple steatosis to NASH has not yet been investigated. Interestingly, it has recently been reported that the strong upregulation of FSP27/CIDEC, with no change to CIDEA and CIDEB expression levels, contributes to alcohol-induced liver damage^19^. We therefore evaluated the expression level of the three CIDE members in NASH development and liver injury in experimental mouse models and in a cohort of patients at various stages of NAFLD progression.

## Results

### Hepatic expression of CIDEA, FSP27α and β increased with obesity-induced hepatic steatosis

**Figure 1:**
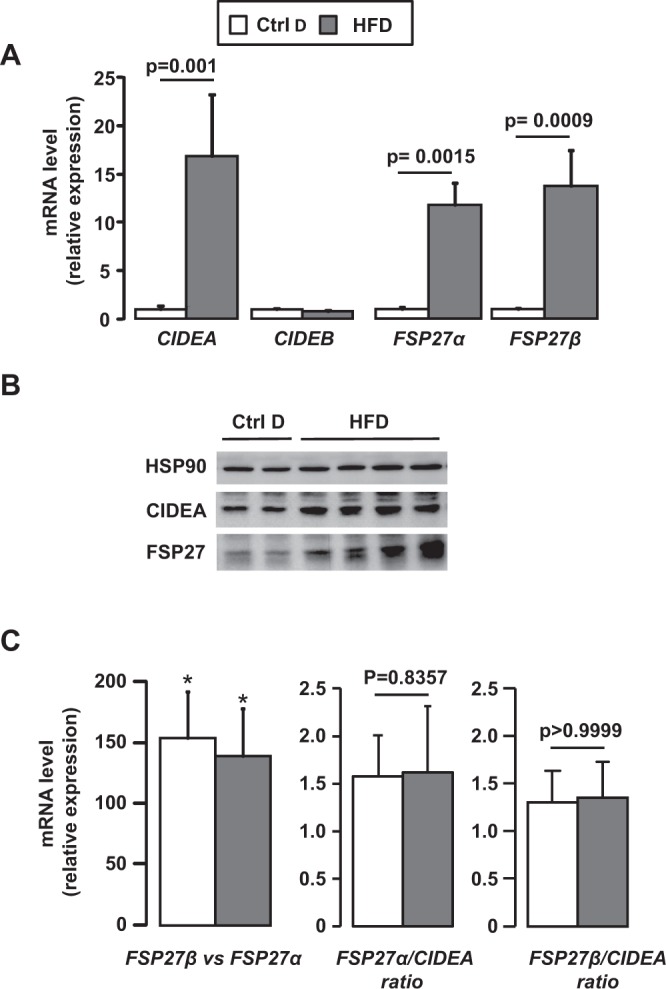
Hepatic expression of CIDEA, FSP27α and FSP27β increased with hepatic steatosis induced by HFD challenge. Wild-type mice fed a control diet (Ctrl D) (n = 7) or HFD (n = 7) for 33 weeks. (**A**,** C**) Hepatic expression of CIDEA, CIDEB, FSP27α and FSB27β was evaluated in Wt, Ctrl D and HFD mice at the mRNA level (7 mice/group). The gene expression was normalized to the mRNA levels of B2M or 36B4. Results are expressed relative to the expression level in controls (means ± SEM) and statistically analyzed using the Mann–Whitney test. (**B**) Hepatic expression of CIDEA, FSP27 and HSP90 was evaluated in Wt, Ctrl D and HFD mice at the protein level (2–4 mice/group). *p < 0.05.

**Figure 2:**
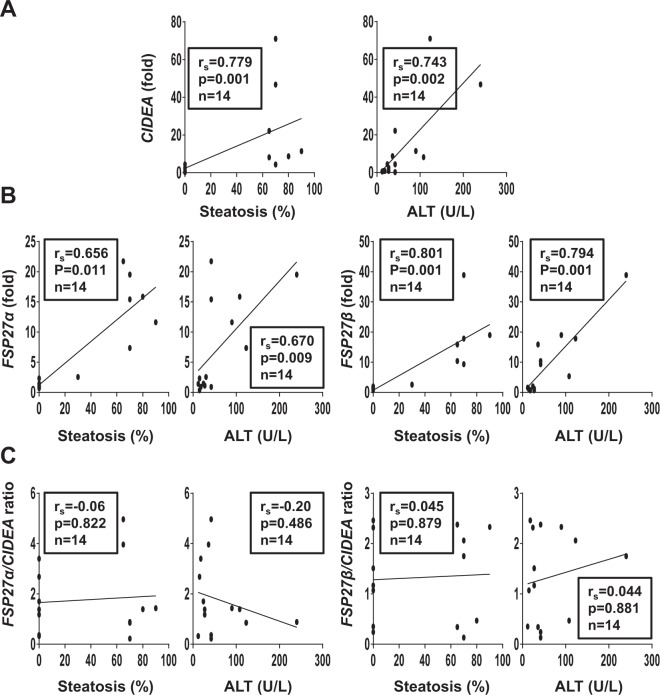
Hepatic CIDEA, FSP27α and FSP27β correlated with hepatic steatosis and liver injury in HFD-induced obese mice. Correlation between hepatic expression of *CIDEA* (**A**), *FSP27α*, *FSB27β* (**B**), FSP27α/CIDEA ratio or FSP27β/CIDEA ratio (**C**) (fold) with hepatic steatosis (%) and ALT in Wt, Ctrl D and HFD mice (7 mice/group, 33 weeks of challenges) were analyzed using the Pearson’s correlation test.

We first evaluated the hepatic expression levels of CIDEA, CIDEB and FSP27α and β in dietary mouse models of obesity and hepatic steatosis. After 33 weeks of high-fat diet (HFD), the wild-type C57BL/6 (Wt) mice developed obesity (Supplementary Fig. 1A), severe hepatic steatosis (Supplementary Fig. 1B,C) and hepatic injury as assessed by ALT activity (Supplementary Fig. 1D). The hepatic expression levels of CIDEA and FSP27 α and β were robustly increased upon 33 weeks of HFD at both the mRNA (Fig. 1A) and protein (CIDEA and FSP27) (Fig. 1B) level. Hepatic expression of *CIDEA*, *FSP27 α and β* also correlated with hepatic steatosis and liver injury (Fig. 2A,B). These occurred at the same extent as evaluated by the *FSP27 α /CIDEA* and *FSP27 β/CIDEA* ratios which did not change with hepatic steatosis and did not correlate with liver injury (Figs 1C, 2C). The hepatic expression of FSP27α and β were similarly increased in obese mice (Fig. 1C: relative expression of FSP27β versus FSP27α). Even though it is robustly expressed in the liver (data not shown), hepatic expression of *CIDEB* did not change with obesity or hepatic steatosis (Fig. 1A). Steatosis and/or obesity were thus associated with the upregulation of the hepatic expression of CIDEA, FSP27α and β.

### Differential expression of CIDEA and FSP27β is related to the severity of the MCDD-mediated steatohepatitis

**Figure 3:**
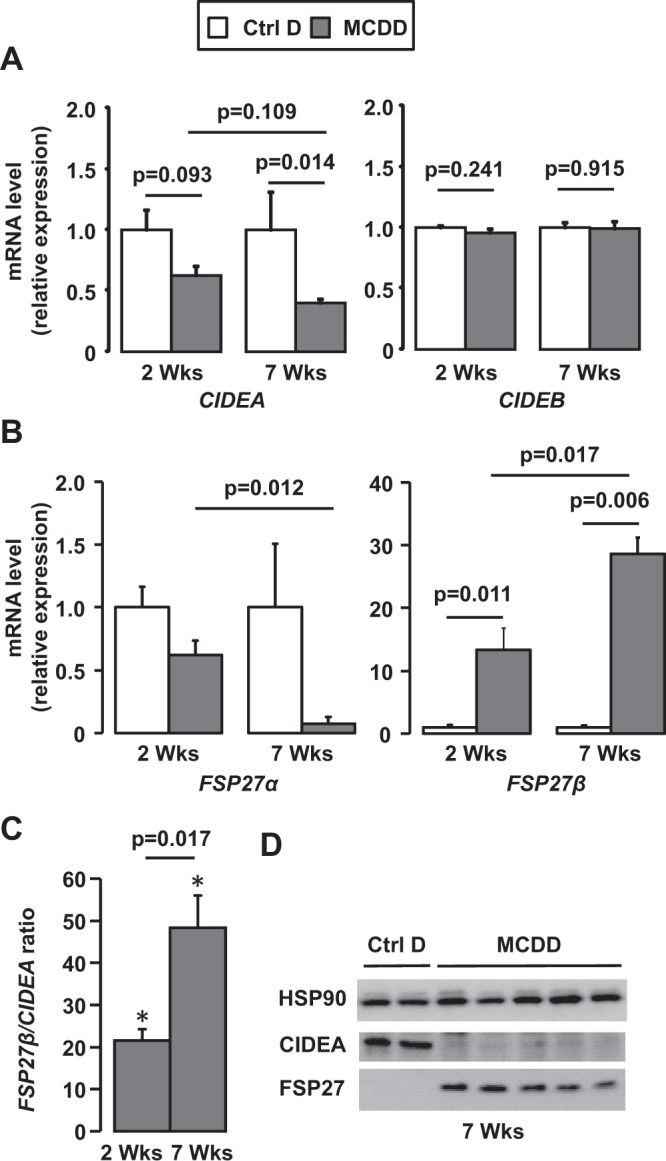
Hepatic expression of FSP27β strongly increased with the severity of NAFLD whereas CIDEA expression decreased. Wild-type mice fed a control diet (Ctrl D) (n = 4) or MCDD (n = 6) for 2 and 7 weeks. (**A**, **B**, **C**) Hepatic expression of CIDEA, CIDEB, FSP27α and FSP27β was evaluated in Wt, Ctrl D and MCDD mice at the mRNA level (4–6 mice/group). The gene expression was normalized to the mRNA levels of B2M or 36B4. Results are expressed relative to the expression level in controls (means ± SEM) and statistically analyzed using the Mann–Whitney test. (**D**) Hepatic expression of CIDEA, FSP27 and HSP90 was evaluated in Wt, Ctrl D and MCDD mice at the protein level (2–5 mice/group). *, versus Ctrl D, p < 0.05

**Figure 4:**
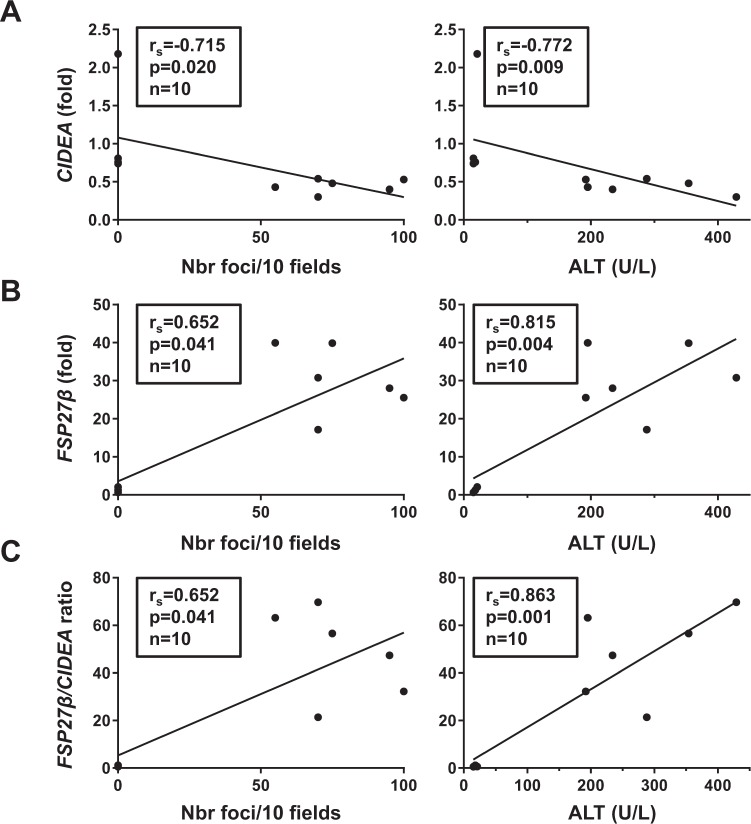
Hepatic CIDEA and FSP27β negatively and positively correlated with steatohepatitis and liver injury in MCDD mice, respectively. Correlation between hepatic expression of *CIDEA* (**A**), *FSP27β* (**B**) or FSP27β/CIDEA ratio (**C**) (fold) with hepatic steatosis (%) and ALT activity (U/L) in Wt, Ctrl D and MCDD mice (4–6 mice/group, 7-week challenge) were analyzed using the Pearson’s correlation test.

To subsequently investigate the behavior of the CIDECs in response to the progression and the severity of the NAFLD, we monitored their hepatic expression in wild-type mice fed a diet deficient in methionine and choline (MCDD) for 2 and 7 weeks. At 2 weeks, mice developed moderate hepatic steatosis with mild hepatic inflammation and liver injury (Supplementary Fig. 2). The long-lasting MCDD challenge (7 weeks) aggravated the liver complications with severe steatosis (from 50 ± 8% at 2 w to 81 ± 3% at 7 w, p = 0.0201), inflammation (from 9 ± 3 foci/10 fields at 2 w to 77 ± 7% at 7 w, p = 0.0024) and liver injury (ALT activity from 51 ± 7 U/L at 2 w to 282 ± 42 U/L at 7 w, p = 0.0024) (Supplementary Fig. 2). Hepatic CIDEA decreased in concert with the severity of the NAFLD at both the mRNA and the protein level (Fig. 3A, D). Its hepatic expression negatively correlated with hepatic inflammation (number of inflammatory foci) and liver injury (ALT activity) after 7 weeks of MCDD (Fig. 4A). In contrast, the hepatic FSP27β was robustly augmented as NAFLD severity increased, from a 13-fold (±3) increase at 2 weeks to a 28-fold (±2) increase after 7 weeks of MCDD (Fig. 3B, D). Hepatic FSP27β also correlated with inflammation and liver injury after 7 weeks of MCDD (Fig. 4B). The increased FSP27 expression was mainly caused by the upregulation of FSP27β since FSP27α expression tended to decrease (Fig. 3B). This inverse regulation pattern for CIDEA and FSP27β expression was better illustrated by the FSP27β/CIDEA ratio, with a 21-fold increase at 2 weeks versus a 48-fold increase at 7 weeks, p = 0.017 (Fig. 3C). This ratio also strongly correlated with hepatic inflammation and liver injury (Fig. 4C). The severity of the steatohepatitis was thus associated with an opposite expression of CIDEA and FSP27β.

### Hepatic CIDEC2 correlated with the severity of the NAFLD in obese patients

**Figure 5:**
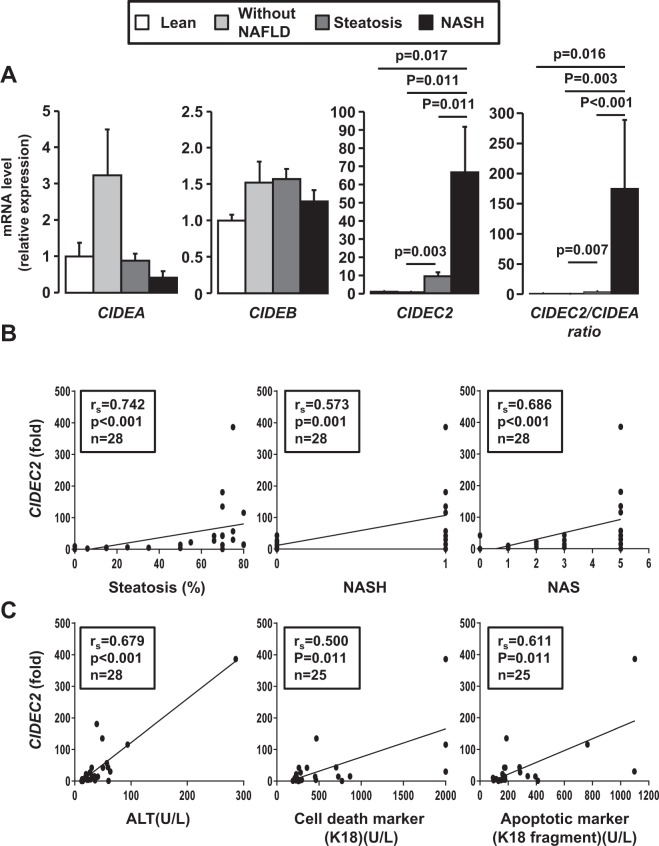
Hepatic CIDEC2 expression progressively increased with steatosis and NASH in obese patients. (**A**) Liver *CIDEA*, *CIDEB* and *CIDEC2* mRNA expression levels were analyzed by real-time quantitative PCR in lean patients (n = 5), in morbidly obese patients without NAFLD (n = 5), with hepatic steatosis (n = 14) and with NASH (n = 9). The gene expression values were normalized to RPLP0 mRNA levels. Results are expressed relative to the expression level in controls (means ± SEM) and statistically analyzed using the Mann–Whitney test. (**B**, **C**) Correlation between hepatic CIDEC2 expression (fold) with hepatic steatosis (%), ALT, NASH and NAFLD activity score (NAS), serum markers of hepatocyte apoptosis (caspases-generated keratin 18 fragment) and hepatocyte death (keratin 18) in 25–28 obese patients were analyzed using the Pearson’s correlation test.

To address the human relevance of our findings, we examined the relationship between hepatic CIDEA, CIDEB, CIDEC1 and CIDEC2 expression and the NAFLD progression from normal liver to steatosis and subsequent NASH in human liver biopsies from morbidly obese patients seeking for bariatric surgery. Patients were classified into 3 groups: without NAFLD, with hepatic steatosis (Steatosis) and with NASH (NASH) (assessed on the basis of three histopathological features steatosis, lobular inflammation and hepatocellular ballooning; Table 1, Supplementary Fig. 3 and Supplementary Methods). Liver mRNA levels of CIDEC2 were progressively upregulated with hepatic steatosis and subsequent NASH (Fig. 5A). This expression correlated with NAFLD features including hepatic steatosis, NASH and NAFLD activity score (NAS)(Fig. 5B). Hepatic CIDEC2 also correlated with hepatic injury as assessed by ALT activity (Fig. 5C) and serum levels of keratin 18 (hepatocyte death marker) and caspase-generated keratin 18 fragment (hepatocyte apoptotic marker)^24,25^ (Fig. 5C). As reported in the mouse model of steatohepatitis, the upregulation of CIDEC is mainly related to CIDEC2 expression since CIDEC1 is not altered (data not shown). In contrast, hepatic CIDEA tended to increase in obesity (lean versus obese patients without NAFLD) and then to decrease in NAFLD (Fig. 5A). This relationship was significantly amplified when the CIDEC2/CIDEA ratio was evaluated (with NAS: r_s_ = 0.861, p < 0.001, n = 26)(Supplementary Fig. 3). Consistent with our animal results, the CIDEB expression was not modified with obesity and NAFLD (Fig. 5A) in human studies.

**Table 1:** Characteristics of 28 morbidly obese patients.

	without NAFLD	with steatosis	with NASH	p
n	5	14	9	
Age (years)	39.4 ± 5.9	37.4 ± 2.4	45.8 ± 3.6	0.186
Sex (F/M)	4/1	14/0	4/5	0.005
BMI (kg/m²)	43.4 ± 0.7	44.1 ± 1.4	43.3 ± 1.8	0.851
ALT (IU/L)	18.20 ± 4.48	26.47 ± 2.95	81.44 ± 26.19^*,#^	<0.001
Insulin level (mIU/L)	7.00 ± 1.10	15.46 ± 3.21	28.67 ± 4.56^*,#^	0.003
Glucose level (mmol/L)	4.88 ± 0.14	5.44 ± 0.13^*^	6.70 ± 0.72^*^	0.014
HOMA-IR	1.52 ± 0.24	3.77 ± 0.80^*^	8.51 ± 1.36^*,#^	0.001
HbA1c (%)	5.34 ± 0.22	5.62 ± 0.12	6.40 ± 0.38^*,#^	0.028
Triglycerides (mmol/L)	1.05 ± 0.15	1.41 ± 0.17	3.42 ± 1.07^*,#^	0.007
HDL cholesterol (mmol/L)	1.53 ± 0.18	1.52 ± 0.10	1.07 ± 0.37^*,#^	0.006
NAFLD Activity Score (n)	0(5)	1(4)/2(4)/3(6)	5(9)	
Grade of steatosis (n)	0(5)	1(4)/2(4)/3(6)	3(9)	
Lobular inflammation (n)	0(5)	0(14)	1(9)	
Hepatocellular ballooning (n)	0(5)	0(14)	1(9)	

Without NAFLD: patients with normal liver histology; Steatosis: patients with steatosis; NASH: patients with severe steatosis and NASH. Data are expressed as mean ± SEM and compared using the non parametric Kruskal-Wallis test (column p) and Mann Whitney test (*, #) for quantitative values and or Khi-deux test for qualitative values. *p < 0.05 compared with “Without NAFLD”. ^#^p < 0.05 compared with “Steatosis”.

### Down regulation of FSP27β resulted in a decreased lipid droplet size in mouse hepatocytes

**Figure 6:**
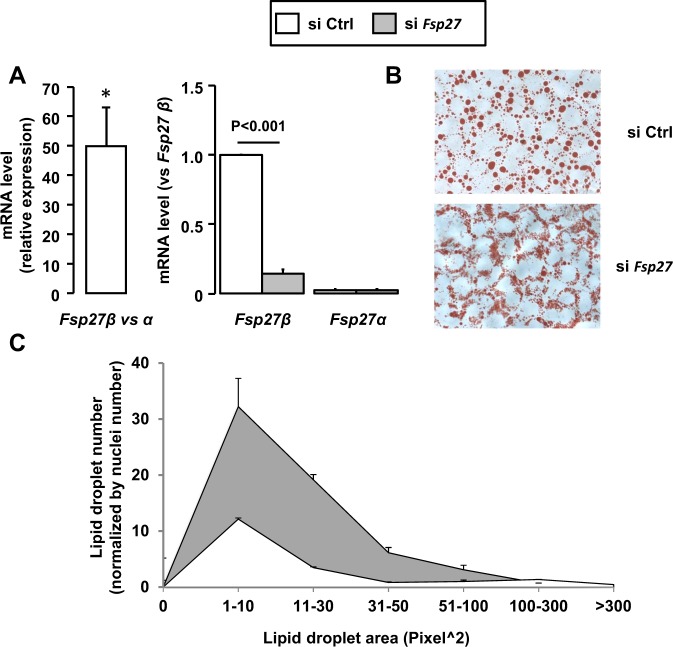
Down regulation of FSP27β resulted in the fractionation of lipid droplets in AML12 hepatocytes. AML12 hepatocytes after control (siCtrl) or FSP27 silencing (si FSP27) were stimulated with oleic acid (0.5 mM) for 16 h (n = 3). (**A**) FSP27α and FSP27β mRNA expression levels were analyzed by real-time quantitative PCR. The gene expression was normalized to RPLP0 mRNA levels. (**B**,**C**) Lipid droplets were stained by Oil-Red O solution for 5 min. (**B**) Representative pictures are shown. (**C**) Lipid droplet areas were quantified (in pixels squared) and normalized according to the number of nuclei on the slides. Results are expressed relative to the control (siCtrl) as means ± SEM. Data were statistically analyzed using the Student’s t-test. *, versus siCtrl, p < 0.05.

Since FSP27β is localized on the surface of lipid droplets and is known to suppress lipolysis^18^, we therefore investigated if altered FSP27β expression could affect hepatocyte lipid droplet synthesis. The down-regulation of FSP27β in AML12 hepatocytes (Fig. 6A), the main isoform expressed in hepatocyte (Fig. 6A), modified the size distribution of the lipid droplets in response to oleic acid (Fig. 6B, C). The number of small lipid droplets increased, while the number of large lipid droplets decreased. FSP27β expression could thus be involved in the development of hepatic macro-steatosis.

### Overexpression of FSP27β sensitized hepatocytes to cell death in response to TNFα and palmitic acid and led to decreased BCL2 expression

**Figure 7:**
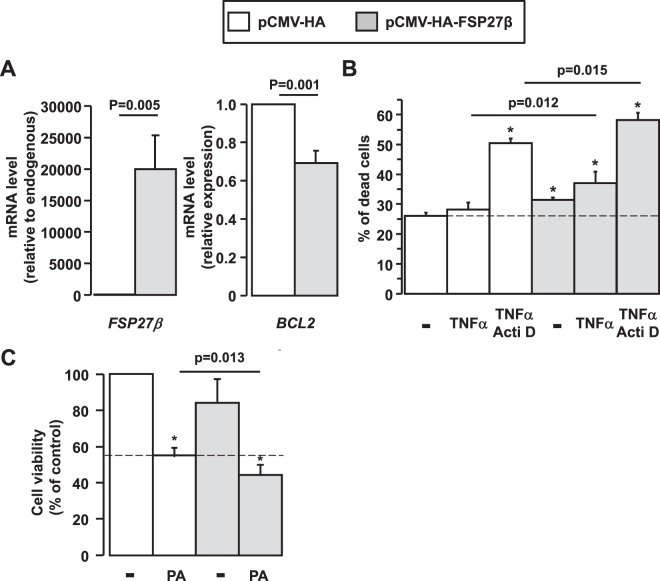
Overexpression of FSP27β enhanced cell death in response to TNFα and palmitic acid in HEPG2 hepatocytes. (**A**) HEPG2 cells were transfected with pCMV-HA and pCMV-HA-FSP27β as indicated. After 48 h, the human *BCL2*, mouse *FSP27* and human *CIDEC* mRNA expression levels were analyzed by real-time quantitative PCR. The gene expression values were normalized to RPLP0 mRNA levels (n = 4). (**B**,**C**) After overexpression of FSP27β with pCMV-HA-FSP27β transfection in HEPG2 cells, (**B**) cell death (flow cytometry)(n = 4) were evaluated in the basal state and in response to TNFα (20 ng/ml) and actinomycin D (0.1 µg/ml) with TNFα (20 ng/ml) for 16 h and (**C**) cell viability (MTT assay)(n = 3), in response to palmitic acid (1 mM) for 16 h. Results relative to the control (pCMV-HA) are expressed as means ± SEM. Data were statistically analyzed using the Student’s t-test. *, versus pCMV-HA p < 0.05.

FSP27, in addition to regulating lipid droplet development, could also mediate apoptosis in an expression-dependent fashion^21,22,26^. Furthermore, our human and experimental data strongly suggested that the expression level of FSP27β (mouse)/CIDEC2 (human) was associated with NAFLD progression and correlated with liver injury in NAFLD (Figs 2, 4 and 5). As TNFα was strongly upregulated in NASH liver, strongly correlated with ALT activity (HFD mice: r_s_ = 0.666, p = 0.007, n = 15; 7 weeks MCDD mice: r_s_ = 0.879, p = 0.002, n = 9) and well reported to mediate hepatocyte death^27–30^, we first evaluated the effect of the FSP27β overexpression on the sensitivity to cell death under basal conditions and in response to TNFα and TNFα with actinomycin D (Acti D). At baseline, the overexpression of FSP27β (Fig. 7A) caused minor increased cell death as evaluated by flow cytometry (Fig. 7B). This slight effect could be explained by the low percentage of transfected cells (20.48 ± 2.84% of GFP^+^ cells; n = 4). Interestingly, this cell death was associated with a decrease in anti-apoptotic *BCL2* expression (Fig. 7A). Furthermore, these effects were further amplified in response to TNFα and TNFα with Acti D in HEPG2 cells overexpressing FSP27β, versus control HEPG2 hepatocytes (Fig. 7B). We then evaluated the effect of the FSP27β overexpression on HEPG2 viability in response to saturated fatty acid (palmitic acid), key player in hepatocyte lipotoxicity. Again, the cell viability was further decreased in response to palmitic acid in HEPG2 cells overexpressing FSP27β, versus control HEPG2 hepatocytes (Fig. 7C). By regulating hepatic steatosis and liver injury, FSP27β thus plays a key role in the NAFLD progression to more severe complications, i.e., NASH.

## Discussion

Here we report differential hepatic expression of CIDEA and FSP27β/CIDEC2 in response to increasing NAFLD severity. Hepatic FSP27β/CIDEC2 expression was not associated with obesity, but progressively increased with hepatic steatosis and subsequent steatohepatitis in mouse and human studies, respectively. In contrast, hepatic CIDEA increased with obesity but its hepatic expression tended to decrease with the severity of the steatohepatitis. The FSP27β/CIDEC2 to CIDEA ratios better illustrated this opposing regulatory pattern and strongly correlated with hepatic inflammation (number of inflammatory foci in mouse studies, and NASH and NAS in patients) and liver injury (ALT activity in mouse and human studies).

As previously demonstrated, the hepatic expression of CIDEA is increased in a mouse model of diet-induced obesity and hepatic steatosis^9,10^. This upregulation could be more closely associated with obesity than hepatic steatosis. Indeed, its hepatic expression tended to increase in obese patients without liver complications and metabolic syndrome, compared to lean patients. It has also been reported that the hepatic expression of CIDEA correlates with body mass index in obese patients^31^. CIDEA polymorphism has also been associated with obesity in human^32,33^, although this could be more closely related to its role in adipose tissue. The role of CIDEA in hepatic triglyceride accumulation and lipid droplet formation has been clearly demonstrated and its targeting (overexpression and down regulation) regulates hepatic steatosis in obese mice^10^. In our cohort of obese patients and in a mouse model of steatohepatitis, the hepatic expression of CIDEA decreased with the severity of liver injury and steatohepatitis. However, it is important to emphasize that this is specific for CIDEA. Indeed, other important elements in the regulation of lipid droplet synthesis, such as PLIN5 and PNPLA2, are still upregulated in the fatty liver tissue of MCDD mice (after 7 weeks of MCDD: 2.3-fold increase for PLIN5, p = 0.0142; 3.13-fold increase for PNPLA2) and strongly correlate with hepatic steatosis in obese patients (PLIN5: r_s_ = 0.698, p < 0.001, n = 30; PNPLA2, r_s_ = 0.767, p < 0.001, n = 30). Since CIDEA expression is mainly dependent on SREBP1c^9,10^ and the down-regulation of SREBP-1c has been associated with the development of burned-out NASH^34^, the decrease in CIDEA could reflect the severity of NAFLD (NASH with fibrosis).

Similarly, the progressive upregulation of FSP27β/CIDEC2 with the progression of NAFLD could be a marker but also an important player. This gradual increase is more closely associated with FSP27β/CIDEC2 than FSP27α/CIDEC1 in mouse and human studies. Xu *et al*., who first described the β isoform of FSP27 in fatty liver, also reported its upregulation in mouse models of diet-induced obesity and steatohepatitis and in human studies^18^. The “mild” upregulation of FSP27β could be related to hepatic steatosis as we and other groups have previously reported (Fig. 1 and^13–17^). Furthermore, we have observed that its down-regulation in hepatic cells promoted the fractionation of lipid droplets in response to oleic acid. In accordance with this, it has been reported that the down-regulation of FSP27β in steatotic liver tissue resulted in reduced accumulation of hepatic triglycerides and lipid droplets^17^.

The strong upregulation of FSP27β/CIDEC2 could be more closely related to steatohepatitis and liver injury. Hepatic expression of FSP27β/CIDEC2 shows a strong correlation with liver injury as evaluated by ALT activity (in mouse and human studies) and a serum marker of hepatocyte apoptosis (in humans). With the increasing severity of the steatohepatitis, hepatic expression of FSP27β rises to a 28-fold (±2) increase in 7 weeks MCDD mice, versus a 13-fold (±3) increase in 2 weeks MCDD and HFD mice. We also observed that overexpression of FSP27β sensitized hepatocytes to cell death mediated by cytokine (TNFα) and saturated fatty acid (palmitic acid). Several groups have reported that FSP27 is a potent apoptotic inducer via the activation of the pro-apoptotic caspases, which trigger both the release of cytochrome c from mitochondria and DNA fragmentation^20–23^. This apoptotic role for FSP27 was found to require the CIDE-C domain. In line with this, overexpression of CIDE-C domain containing FSP27α also sensitized hepatocytes to cell death mediated by cytokine (TNFα) (Supplementary Fig. 4), while FSP27α expression tended to decrease with the severity of the steatohepatitis. Interestingly, the same domain of FSP27 also mediated its localization to lipid droplets and its interaction with CIDEA^21,35^. The inverse patterns of regulation for the hepatic expression of CIDEA (down-regulated) and FSP27β (up-regulated) is robustly amplified with the NAFLD progression (FSP27β/CIDEA ratio ranges from 1.6 ± 0.6 in simple steatosis (HFD mice) to 21 ± 2 in steatohepatitis (2 weeks MCDD mice) and 48 ± 7 with advanced steatohepatitis (7 weeks MCDD mice)). This opposing regulatory pattern could also modify the role of the C domain of FSP27β, which could be more involved in the alteration of lipid droplets or the initiation of the apoptotic program, rather than binding with CIDEA.

Interestingly, the role of FSP27/CIDEC has recently been investigated in alcoholic steatohepatitis. Xu *et al*. have reported that the elevation of FSP27 (both isoforms with FSP27β 1000-fold higher than FSP27α) was likely to induce steatosis and liver injury in mouse model of chronic plus binge ethanol feeding. In this mouse model, the expression of CIDEA and CIDEB was not modified, hepatic FSP27 was strongly upregulated and the down regulation of FSP27 in hepatocytes prevented liver injury combined with the mitochondrial production of ROS. The authors also reported that hepatic expression of CIDEC mRNA increased more than 40-fold in samples from patients with alcoholic hepatitis and correlated with severity of the disease^19^. The authors further reported that the sustained FSP27 elevation in hepatocytes likely causes chronic liver injury and inflammation, and may subsequently induce fibrosis in a mouse model that combines chronic and acute-on chronic liver injury^19^.

Finally, we also report that the hepatic expression of CIDEB (at the mRNA level) is independent of obesity, as previously reported^31,36^, and also independent of hepatic steatosis and steahepatitits in mouse and human studies. While the level of gene expression was stable, CIDEB was the most highly expressed among the 3 members of the CIDE family in hepatocytes (2000x more than CIDEA and 400x more than FSP27/CIDEC in mice and humans) and could be an important player in the development of hepatic steatosis since it regulates the formation of triacylglycerol-enriched VLDL particles^36^.

In this current study, we demonstrated that the hepatic expressions of the members of the CIDE family are differentially regulated according to the development and the severity of NAFLD in mouse and human studies. The gradual elevation of FSP27β/CIDEC2 expression with the development of hepatic steatosis and subsequent steatohepatitis reinforces our interest in this protein as a potential therapeutic target. It would need to be targeted only when robustly upregulated, and in a hepatocyte-specific manner in order to maintain its beneficial role in the storage of lipids in adipocytes^37^.

## Material and Methods

### Human samples

In this study, human samples (blood, liver biopsy) were from twenty-eight morbidly obese patients and five lean subjects. All information relative to the patients are described in Supplementary Methods, Table 1 and Ethics approval section.

### Mice

Male mice were used in all experiments described in Supplementary Methods. All animal experiments were approved by the CIEPAL and Use committee.

Real-time quantitative PCR analysis has been performed as previously described^38,39^ and described in the Supplementary Methods.

### Immunoblotting

Cells or frozen tissues were solubilized in lysis buffer (20 mM Tris, pH 7.4, 150 mM NaCl, 10 mM EDTA, 150 mM NaF, 2 mM sodium orthovanadate, 10 mM pyrophosphate, proteases inhibitors cocktail, and 1% Triton X-100) for 45 min at 4 °C. Lysates were cleared (14 000 rpm, 15 min). Proteins were quantified (BCA Protein assay kit, 23225 Thermo Fisher Scientific Inc.), separated by SDS-PAGE and immunoblotted as previously described^38^. The proteins were probed with anti-CIDEA (NBP1-76950, Novus Biologicals), anti-FSP27 (ab77115, ab198204 Abcam), and anti-HSP90 (#4877, Cell Signaling) antibodies at 1 µg/mL.

### Cellular models and treatments

#### *Down regulation of FSP27*

Down regulation of FSP27 was achieved using ON-TARGET plus SMART pool technologies (L-040997, Mouse FSP27, NM_178373 or non-targeting siRNA as a control, Darmacon, CO) and Lipofectamine RNAiMAX technologies (MSS236551, Mouse FSP27 Life Technologies) in AML-12 hepatocytes (ATCC, CRL-2254). Following a 24-hour transfection, oleic acid (0.5 mM) was applied to cells for 16 hours. Cells were then rinsed twice with PBS and fixed with 4% formaldehyde solution. Lipid droplets were stained by Oil-Red O solution for 5 min. Microscope slides were prepared (x60 magnification) for lipid droplet quantification. The area of the lipid droplets was quantified (in pixels squared) and normalized by the number of nuclei on the slides.

#### *Overexpression of FSP27β*

HEPG2 cells (ATCC HB-8065) were transfected with pCMV-HA FSP27β or the empty plasmid (provided by Drs Xu Xu and Ann-Hwee Lee^18^) using a Jet PEI-hepatocyte mix assay (102-05NOzyme). After 48 h, cells were treated with TNFα (20 ng/ml), actinomycin D (0.1 µg/ml) with TNFα (20 ng/ml) or palmitic acid (1 mM) for 16 h. Gene expression, cell viability and cell death were then evaluated as indicated.

### MTT assay

The assay is dependent on the ability of viable cells to metabolize a water-soluble tetrazolium salt into a water-insoluble formazan product. Following the indicated treatments, cells were incubated for 2 h with 0.5 mg/mL MTT (3-(4,5-Dimethylthiazol-2-yl)-2,5-Diphenyltetrazolium Bromide) in serum-free medium (DMEM). After removing the supernatant, DMSO was added to completely dissolve the formazan product. Aliquots of the resulting solutions were transferred to 96-well plates and the absorbance was recorded at 550 nm using the microplate spectrophotometer system (ELX800, Bio-TEK instruments). Results are presented as a percentage of the control values.

### Cell death

Flow cytometry was used to evaluate cell death following double staining with annexin-V-PE and 7-AAD according to the manufacturer’s instructions (Annexin V-PE apoptosis detection kit I, BD Biosciences, Pont de claix, France).

### Statistical analysis

Statistical significance between two human or mouse study groups was determined using the nonparametric Mann–Whitney test. Data from cell lines were statistically analyzed using the Student t-test. Pearson’s correlation test has been used for the correlative analysis. Significance has been considered for P < 0.05.

### Ethics approval

All subjects gave their informed written consent to participate in this study in accordance with French legislation regarding Ethics and Human Research (Huriet-Serusclat law). The “Comité Consultatif de Protection des Personnes dans la Recherche Biomédicale de Nice” approved the study (07/04:2003, N° 03.017). The guidelines of laboratory animal care were followed. The local CIEPAL committee (Comité Institutionel d’Ethique Pour l’Animal de Laboratoire, national agrement n° 28) has approved the animal experiments (NCE/2013-108, APAFIS#51 00–20 15121 1 10477413 v6). (Authorization of the C3M animal facility: B06-088-20).

## Supplementary information

Supplementary data and methods, unmodified gels

## References

[1] Younossi ZM (2016). Global epidemiology of nonalcoholic fatty liver disease-Meta-analytic assessment of prevalence, incidence, and outcomes. Hepatology.

[2] Samuel VT, Shulman GI (2018). Nonalcoholic Fatty Liver Disease as a Nexus of Metabolic and Hepatic Diseases. Cell Metab.

[3] Raff EJ (2015). Diabetes Mellitus Predicts Occurrence of Cirrhosis and Hepatocellular Cancer in Alcoholic Liver and Non-alcoholic Fatty Liver Diseases. J Clin Transl Hepatol.

[4] Lallukka S, Yki-Jarvinen H (2016). Non-alcoholic fatty liver disease and risk of type 2 diabetes. Best Pract Res Clin Endocrinol Metab.

[5] Targher G, Day CP, Bonora E (2010). Risk of cardiovascular disease in patients with nonalcoholic fatty liver disease. N Engl J Med.

[6] Listenberger LL (2003). Triglyceride accumulation protects against fatty acid-induced lipotoxicity. Proc Natl Acad Sci USA.

[7] Yamaguchi K (2007). Inhibiting triglyceride synthesis improves hepatic steatosis but exacerbates liver damage and fibrosis in obese mice with nonalcoholic steatohepatitis. Hepatology.

[8] Minehira, K. & Gual, P. Role of Lipid Droplet Proteins in the Development of NAFLD and Hepatic Insulin Resistance. *In: Valenzuela Baez R* (*eds*) *Non-Alcoholic Fatty Liver Disease* IntechOpen, 55–77, 10.5772/intechopen.71572 (2018).

[9] Wang R (2010). Sterol-regulatory-element-binding protein 1c mediates the effect of insulin on the expression of Cidea in mouse hepatocytes. Biochem J.

[10] Zhou L (2012). Cidea promotes hepatic steatosis by sensing dietary fatty acids. Hepatology.

[11] Li JZ (2007). Cideb regulates diet-induced obesity, liver steatosis, and insulin sensitivity by controlling lipogenesis and fatty acid oxidation. Diabetes.

[12] Li X (2012). Opposing roles of cell death-inducing DFF45-like effector B and perilipin 2 in controlling hepatic VLDL lipidation. J Lipid Res.

[13] Aibara D (2013). Expression of hepatic fat-specific protein 27 depends on the specific etiology of fatty liver. Biol Pharm Bull.

[14] Uno K (2012). Hepatic peroxisome proliferator-activated receptor-gamma-fat-specific protein 27 pathway contributes to obesity-related hypertension via afferent vagal signals. Eur Heart J.

[15] Flach RJ, Qin H, Zhang L, Bennett AM (2011). Loss of mitogen-activated protein kinase phosphatase-1 protects from hepatic steatosis by repression of cell death-inducing DNA fragmentation factor A (DFFA)-like effector C (CIDEC)/fat-specific protein 27. J Biol Chem.

[16] Yu S (2003). Adipocyte-specific gene expression and adipogenic steatosis in the mouse liver due to peroxisome proliferator-activated receptor gamma1 (PPARgamma1) overexpression. J Biol Chem.

[17] Matsusue K (2008). Hepatic steatosis in leptin-deficient mice is promoted by the PPARgamma target gene Fsp27. Cell Metab.

[18] Xu X, Park JG, So JS, Lee AH (2015). Transcriptional activation of Fsp27 by the liver-enriched transcription factor CREBH promotes lipid droplet growth and hepatic steatosis. Hepatology.

[19] Xu MJ (2015). Fat-Specific Protein 27/CIDEC Promotes Development of Alcoholic Steatohepatitis in Mice and Humans. Gastroenterology.

[20] Inohara N, Koseki T, Chen S, Wu X, Nunez G (1998). CIDE, a novel family of cell death activators with homology to the 45 kDa subunit of the DNA fragmentation factor. EMBO J.

[21] Liu K (2009). Functional analysis of FSP27 protein regions for lipid droplet localization, caspase-dependent apoptosis, and dimerization with CIDEA. Am J Physiol Endocrinol Metab.

[22] Tang X, Xing Z, Tang H, Liang L, Zhao M (2011). Human cell-death-inducing DFF45-like effector C induces apoptosis via caspase-8. Acta Biochim Biophys Sin (Shanghai).

[23] Yonezawa T, Kurata R, Kimura M, Inoko H (2011). Which CIDE are you on? Apoptosis and energy metabolism. Mol Biosyst.

[24] Anty R (2010). A new composite model including metabolic syndrome, alanine aminotransferase and cytokeratin-18 for the diagnosis of non-alcoholic steatohepatitis in morbidly obese patients. Aliment Pharmacol Ther.

[25] Lavallard VJ (2011). Serum markers of hepatocyte death and apoptosis are non invasive biomarkers of severe fibrosis in patients with alcoholic liver disease. PLoS One.

[26] Kim JY (2008). Assessment of fat-specific protein 27 in the adipocyte lineage suggests a dual role for FSP27 in adipocyte metabolism and cell death. Am J Physiol Endocrinol Metab.

[27] Crespo J (2001). Gene expression of tumor necrosis factor alpha and TNF-receptors, p55 and p75, in nonalcoholic steatohepatitis patients. Hepatology.

[28] Feldstein AE, Gores GJ (2005). Apoptosis in alcoholic and nonalcoholic steatohepatitis. Front Biosci.

[29] Ribeiro PS (2004). Hepatocyte apoptosis, expression of death receptors, and activation of NF-kappaB in the liver of nonalcoholic and alcoholic steatohepatitis patients. Am J Gastroenterol.

[30] Bertola A (2010). Hepatic expression patterns of inflammatory and immune response genes associated with obesity and NASH in morbidly obese patients. PLoS One.

[31] Hall AM, Brunt EM, Klein S, Finck BN (2010). Hepatic expression of cell death-inducing DFFA-like effector C in obese subjects is reduced by marked weight loss. Obesity (Silver Spring).

[32] Dahlman I (2005). The CIDEA gene V115F polymorphism is associated with obesity in Swedish subjects. Diabetes.

[33] Zhang L, Miyaki K, Nakayama T, Muramatsu M (2008). Cell death-inducing DNA fragmentation factor alpha-like effector A (CIDEA) gene V115F (G– > T) polymorphism is associated with phenotypes of metabolic syndrome in Japanese men. Metabolism.

[34] Nagaya T (2010). Down-regulation of SREBP-1c is associated with the development of burned-out NASH. J Hepatol.

[35] Jambunathan S, Yin J, Khan W, Tamori Y, Puri V (2011). FSP27 promotes lipid droplet clustering and then fusion to regulate triglyceride accumulation. PLoS One.

[36] Ye J (2009). Cideb, an ER- and lipid droplet-associated protein, mediates VLDL lipidation and maturation by interacting with apolipoprotein B. Cell Metab.

[37] Rajamoorthi A, Arias N, Basta J, Lee RG, Baldan A (2017). Amelioration of diet-induced steatohepatitis in mice following combined therapy with ASO-Fsp27 and fenofibrate. J Lipid Res.

[38] Patouraux S (2017). CD44 is a key player in non-alcoholic steatohepatitis. J Hepatol.

[39] Patouraux S (2014). Osteopontin deficiency aggravates hepatic injury induced by ischemia-reperfusion in mice. Cell Death Dis.

