# Supplementary material for: The Differential Expression of Cide Family Members is Associated with Nafld Progression from Steatosis to Steatohepatitis
Source: Sci Rep. 2019 May 16;9:7501. doi: 10.1038/s41598-019-43928-7 (PMC6522528; doi:10.1038/s41598-019-43928-7)

THE DIFFERENTIAL EXPRESSION OF CIDE FAMILY MEMBERS IS ASSOCIATED WITH NAFLD PROGRESSION FROM STEATOSIS TO STEATOHEPATITIS.

Arnaud Sans[1,2 $]; Stéphanie Bonnafous[1,2 $]; Déborah Rousseau[1 $]; Stéphanie Patouraux[1,2]; Clémence M Canivet[1,2]; Pierre S Leclere[1]; Jeanne Tran-Van-Nhieu[3]; Carmelo Luci[1]; Béatrice Bailly-Maitre[1]; Xu Xu[4]; Ann-Hwee Lee[5]; Kaori Minehira[6]; Rodolphe Anty[1,2]; Albert Tran[1,2]; Antonio Iannelli[1,2]; Philippe Gual[1 *].

[1] Université Côte d'Azur, INSERM, U1065, C3M, France;

[2] Université Côte d'Azur, CHU, INSERM, U1065, C3M, France

[3] HU Henri Mondor, Department of Pathology, AP-HP - Université Paris Est Créteil, Créteil, France;

[4] Weill Cornell Medicine, Department of Medicine, Division of Gastroenterology and Hepatology, New York, US

[5] Department of Pathology and Laboratory Medicine, Weill Cornell Medical College, New York, US

[6] University of Lausanne, Department of Physiology, Lausanne, Switzerland

$ These authors participated equally to this work.

* *Corresponding Author:* Philippe Gual, INSERM U1065, Bâtiment Universitaire ARCHIMED, Team 8 "Chronic liver diseases associated with obesity and alcohol", 151 route Saint Antoine de Ginestière, BP 2 3194, 06204 Nice Cedex 03, FRANCE, philippe.gual@inserm.fr, Phone: +33-4-89 06 42 95, Fax: +33-4-89 06 42 21

Supplementary Methods

Human studies. **Morbidly obese patients:** (n=28) were recruited through the Department of Digestive Surgery and Liver Transplantation (Archet 2, University Hospital, Nice, France) where they underwent bariatric surgery for their morbid obesity. Bariatric surgery was indicated for these patients in accordance with French guidelines. Exclusion criteria were: presence of hepatitis B or hepatitis C infection, excessive alcohol consumption (>20g/d) or another cause of chronic liver disease as previously described [1-3]. The characteristics of the study groups are described in Table 1. Before surgery, fasting blood samples were obtained and used to measure alanine and aspartate transaminases (ALT and AST, respectively), glucose, insulin and HbA1c. Insulin resistance was calculated using the homeostatic model assessment (HOMA-IR) index [4]**.** Surgical liver biopsies were obtained during surgery and no ischemic preconditioning was performed. Hepatic histopathological analysis was performed according to the scoring system of Kleiner et al. [5]**.** Three histopathological features were semi-quantitatively evaluated: grade of steatosis (0, <5%; 1, 5%-30%; 2, >30%-60%; 3, >60%), lobular inflammation (0, no inflammatory foci; 1, <2 inflammatory foci per 200x field; 2, 2-4 inflammatory foci per 200x field; 3, >4 inflammatory foci per 200x field) and hepatocellular ballooning (0, none; 1, few balloon cells; 2, many cells/prominent ballooning). **Control subjects**: liver tissue was obtained from 5 lean subjects (5 women; age, 44±9 years; BMI, 21±1.9 kg/m²) undergoing partial hepatectomy for benign tumors (neighbour tissues from four adenoma and one focal nodular hyperplasia)(HU Henri Mondor, Department of Pathology, AP-HP - Université Paris Est Créteil, Créteil, France). Three subjects underwent a left lobectomy or a bisegmentectomy without ischemic preconditioning and two patients underwent a right hepatectomy with a potential ischemic preconditioning (no data). Liver samples did not display any hepatic steatosis, inflammation or fibrosis.

Animals and study design. HFD: 7-week-old wild-type (Wt) mice (Janvier-Labs) were acclimated to our animal facilities under a 12/12h light/dark cycle at a temperature of 21 ± 2 °C and were fed *ad libitum* either a high-fat diet (HFD: 45% fat D12451; Research Diet) or control diet (Ctrl D) for 33 weeks. **MCDD:**19-week-old male wild-type (Wt) C57BL/6 male mice (Janvier-Labs) were acclimated to our animal facilities under a 12/12h light/dark cycle at a temperature of 21 ± 2 °C and were fed *ad libitum* either a methionine- and choline-deficient diet (MCDD) or control diet (Ctrl D) for 2 or 7 weeks (diets from SSNIFF (MCDD # E15653-94; ctr diet # E15654-04). At the end of the corresponding challenge, blood was collected and mice were immediately sacrificed, after which the liver was removed. One part of the liver was immediately frozen in liquid nitrogen and stored at -80°C until analysis. The second part was fixed in buffered formalin, paraffin-embedded, sectioned, and stained with Hematoxylin-Eosin-Saffron. The guidelines of laboratory animal care were followed, and the local ethical committee approved the animal experiments (CIEPAL: Comité Institutionel d'Ethique Pour l'Animal de Laboratoire, national agrement n° 28)(NCE/2013-108, APAFIS#51 00-20 15121 1 10477413 v6). (Authorization of the C3M animal facility: B06-088-20)

Real-time quantitative PCR analysis: Total liver RNA was extracted using the RNeasy Mini Kit (74104, Qiagen, Hilden, Germany) and treated with Turbo DNA-free DNase (AM 1907, Thermo Fisher Scientific Inc.) following the manufacturer's protocol. The quantity and quality of the RNA samples were determined using the Agilent 2100 Bioanalyzer with RNA 6000 Nano Kit (5067-1511, Agilent Technologies, Santa Clara, CA, USA). Total RNA (1 µg) was reverse transcribed with the High-Capacity cDNA Reverse Transcription Kit (Thermo Fisher Scientific Inc.). Real-time quantitative PCR was performed in duplicate for each sample using the StepOne Plus Real-Time PCR System (Thermo Fisher Scientific Inc.) as previously described [6,7]. TaqMan gene expression assays were purchased from Thermo Fisher Scientific Inc.: *RPLP0 (Hs99999902_m1); B2M (Mm00437762_m1); CIDEA (Hs00154455_m1, Mm00432554_m1); CIDEB (Hs00205339_m1,*

Mm00438213_m1);; BCL2 (Hs00153350_m1). Gene expression was normalized to the β2M (β2 microglobulin, mouse) or RPLP0 (Ribosomal Phosphoprotein Large P0, mouse and human) housekeeping genes and calculated based on the comparative cycle threshold Ct method ($2^{-\Delta\Delta Ct}$). The FSP27α and β amplifications were performed using Fast SYBR™ Green technology (Thermo Fisher Scientific Inc.) with the following primers (purchased from Eurogentec: 49001 Angers, FRANCE) mouse Fsp27 alpha Forward GCC-ACG-CGG-TAT-TGC-CAG-GA; mouse Fsp27 beta Forward GTG-ACC-ACA-GCT-TGG-GTC-GGA*;* mouse Fsp27 common Reverse GGG-TCT-CCC-GGC-TGG-GCT-TA*;* human CIDEC1 forward GCA-GTA-TTT-CCA-GGA-GGC-TGT; human CIDEC2 Forward CCA-GAG-CCA-GGG-GAT-GAG-AA; human CIDE common Reverse TGG-AGA-GGG-ACT-TGG-GGT-AG as previously described [8]. Gene expression was normalized to the RPLP0/36B4 housekeeping gene (mouse and human) and calculated based on the comparative cycle threshold Ct method ($2^{-\Delta\Delta Ct}$). Human RPLP0 Forward TGC-ATC-AGT-ACC-CCA-TTC-TAT-CAT; human RPLP0 Reverse AG-GTG-TAA-TCC-GTC-TCC-ACA-GA; mouse 36B4 Forward TCC-AGG-CTT-TGG-GCA-TCA; mouse 36B4 Reverse CTT-TAT-CAG-CTG-CAC-ATC-ACT-CAG-A.

1 Bekri, S. *et al.* Increased adipose tissue expression of hepcidin in severe obesity is independent from diabetes and NASH. *Gastroenterology* **131**, 788-796 (2006).

2 Anty, R. *et al.* The inflammatory C-reactive protein is increased in both liver and adipose tissue in severely obese patients independently from metabolic syndrome, Type 2 diabetes, and NASH. *Am J Gastroenterol* **101**, 1824-1833 (2006).

3 Bertola, A. *et al.* Identification of adipose tissue dendritic cells correlated with obesity-associated insulin-resistance and inducing th17 responses in mice and patients. *Diabetes* **61**, 2238-2247, doi:db11-1274 [pii]10.2337/db11-1274 (2012).

4 Wallace, T. M., Levy, J. C. & Matthews, D. R. Use and abuse of HOMA modeling. *Diabetes Care* **27**, 1487-1495 (2004).

5 Kleiner, D. E. *et al.* Design and validation of a histological scoring system for nonalcoholic fatty liver disease. *Hepatology* **41**, 1313-1321 (2005).

6 Patouraux, S. *et al.* Osteopontin deficiency aggravates hepatic injury induced by ischemia-reperfusion in mice. *Cell Death Dis* **5**, e1208, doi:10.1038/cddis.2014.174cddis2014174 [pii] (2014).

7 Patouraux, S. *et al.* The osteopontin level in liver, adipose tissue and serum is correlated with fibrosis in patients with alcoholic liver disease. *PLoS One* **7**, e35612, doi:10.1371/journal.pone.0035612PONE-D-11-16579 [pii] (2012).
8 Xu, X., Park, J. G., So, J. S. & Lee, A. H. Transcriptional activation of Fsp27 by the liver-enriched transcription factor CREBH promotes lipid droplet growth and hepatic steatosis. *Hepatology* **61**, 857-869, doi:10.1002/hep.27371 (2015).

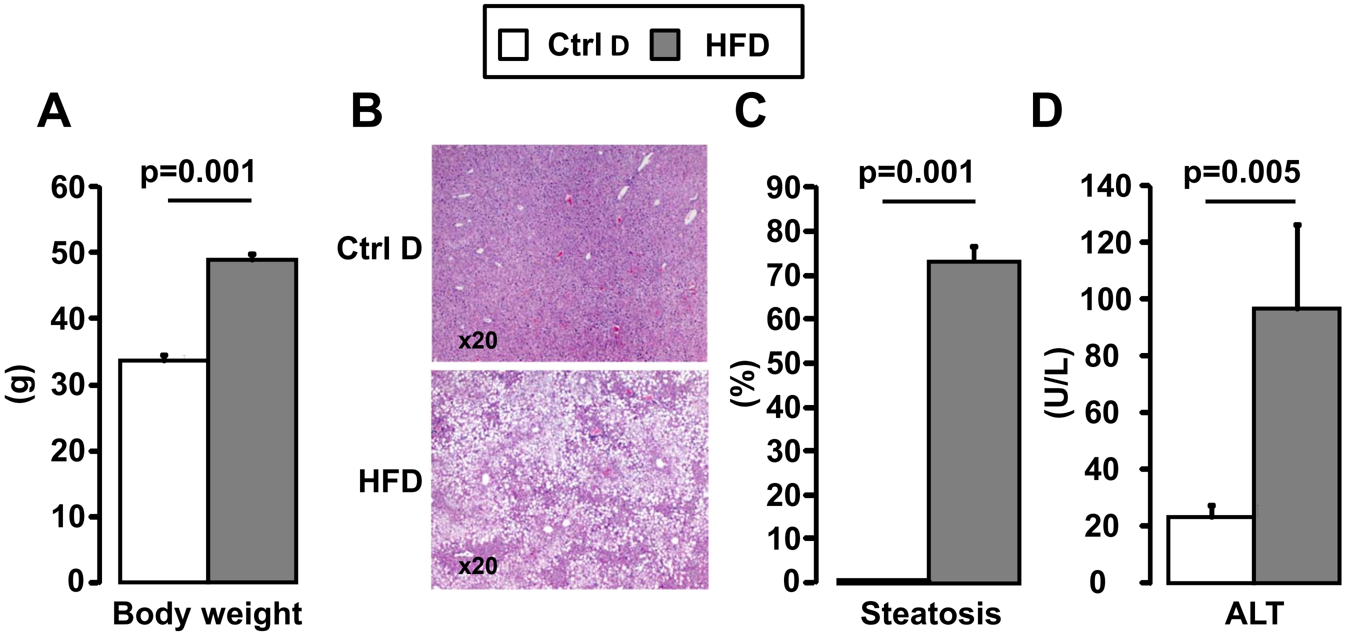

Supplementary Figure 1 : Liver complications in response to 33 weeks of HFD challenge. Wild-type mice fed a control diet (Ctrl D) (n=7) or HFD (n=7) for 33 weeks. **(A)** Body weight after challenge. (**B**) H&E staining of liver tissue section samples from Wt mice after Ctrl D and HFD as indicated. Representative pictures are shown. (**C**) Quantification of hepatic steatosis. (**D**) The plasma levels of ALT were evaluated (7 mice/group). Results are expressed relative to the expression level in controls (means±SEM) and statistically analyzed using the Mann–Whitney test.

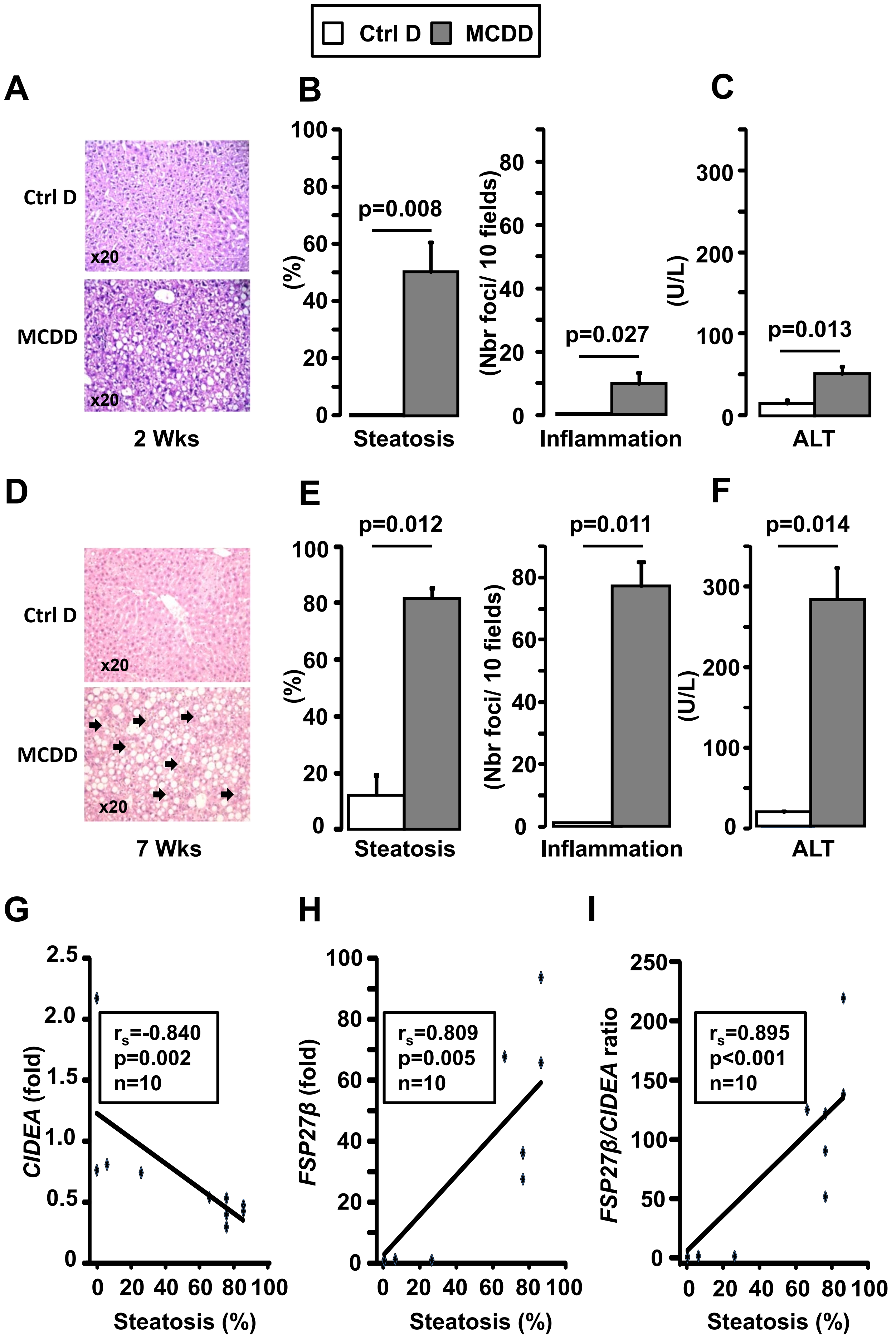

Ctrl D
MCDD
A
B
C
Ctrl D
MCDD
x20
x20
2 Wks
(%)
p=0.008
Steatosis
(Nbr foci/ 10 fields)
p=0.027
Inflammation
(U/L)
p=0.013
ALT
D
E
F
Ctrl D
MCDD
x20
x20
7 Wks
p=0.012
Steatosis
p=0.011
Inflammation
p=0.014
ALT
G
H
I
CIDEA (fold)
r$_s$=-0.840
p=0.002
n=10
Steatosis (%)
FSP27β (fold)
r$_s$=0.809
p=0.005
n=10
Steatosis (%)
FSP27β/CIDEA ratio
r$_s$=0.895
p<0.001
n=10
Steatosis (%)

Supplementary Figure 2 : Liver complications in response to 2 and 7 weeks of MCDD. Wild-type mice fed a control diet (Ctrl D) (4 mice/group) or MCDD (6 mice/group) for 2 **(A-C)** and 7 **(D-F)** weeks. **(A, D)** H&E staining of liver tissue sections samples from Wt mice after Ctrl D and MCDD as indicated. Representative pictures are shown. (**B, E**) Quantification of hepatic steatosis and inflammatory foci. (**C, F**) The plasma levels of ALT were evaluated (4-6 mice/group). Results are expressed relative to the expression level in controls (means±SEM) and statistically analyzed using the Mann–Whitney test. **(G-I)** Correlation between hepatic expression of *CIDEA* **(G)**, *FSP27β* **(H)** or *FSP27β/CIDEA* ratio **(I)** (fold) with hepatic steatosis (%) in Wt, Ctrl D and MCDD mice (4-6 mice/group, 7 weeks of challenge) were analyzed using the Pearson's correlation test.

A

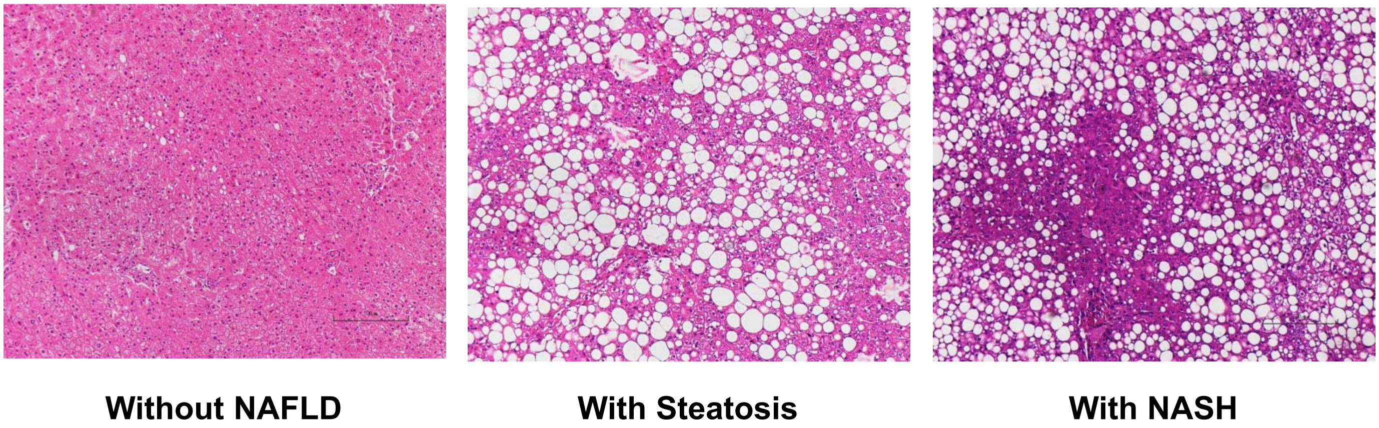

B

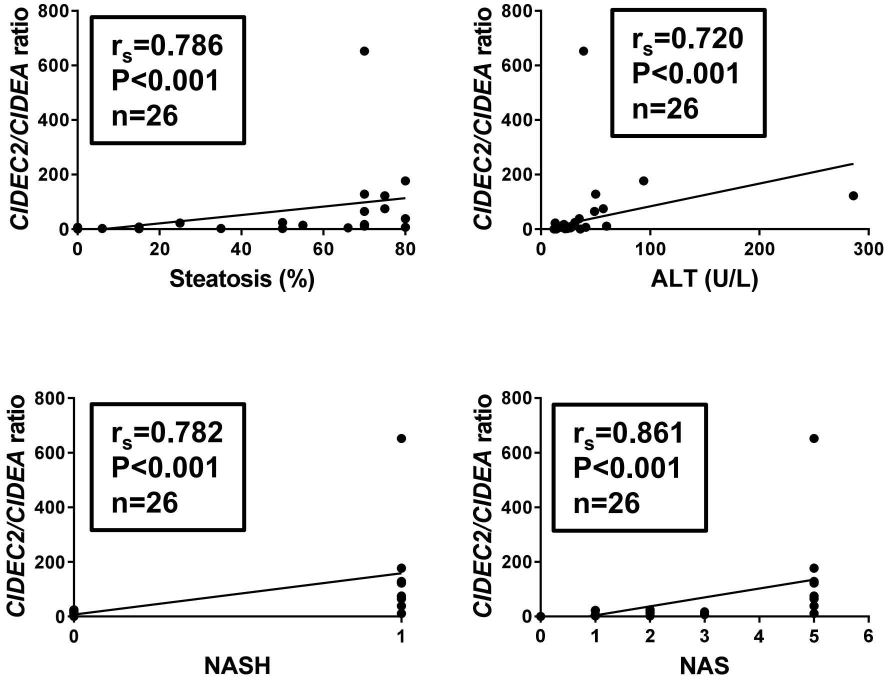

Supplementary Figure 3: A) Liver histology analysis of representative morbidly obese patients without NAFLD, with hepatic steatosis and with NASH (HES staining, x10). B) Hepatic *CIDEC2/CIDEA* ratio correlated with NAFLD features in obese patients. Correlation between hepatic *CIDEC2* expression (fold) with hepatic steatosis (%), ALT activity, NASH and NAFLD activity score (NAS) in 26 obese patients were analyzed using the Pearson's correlation test.

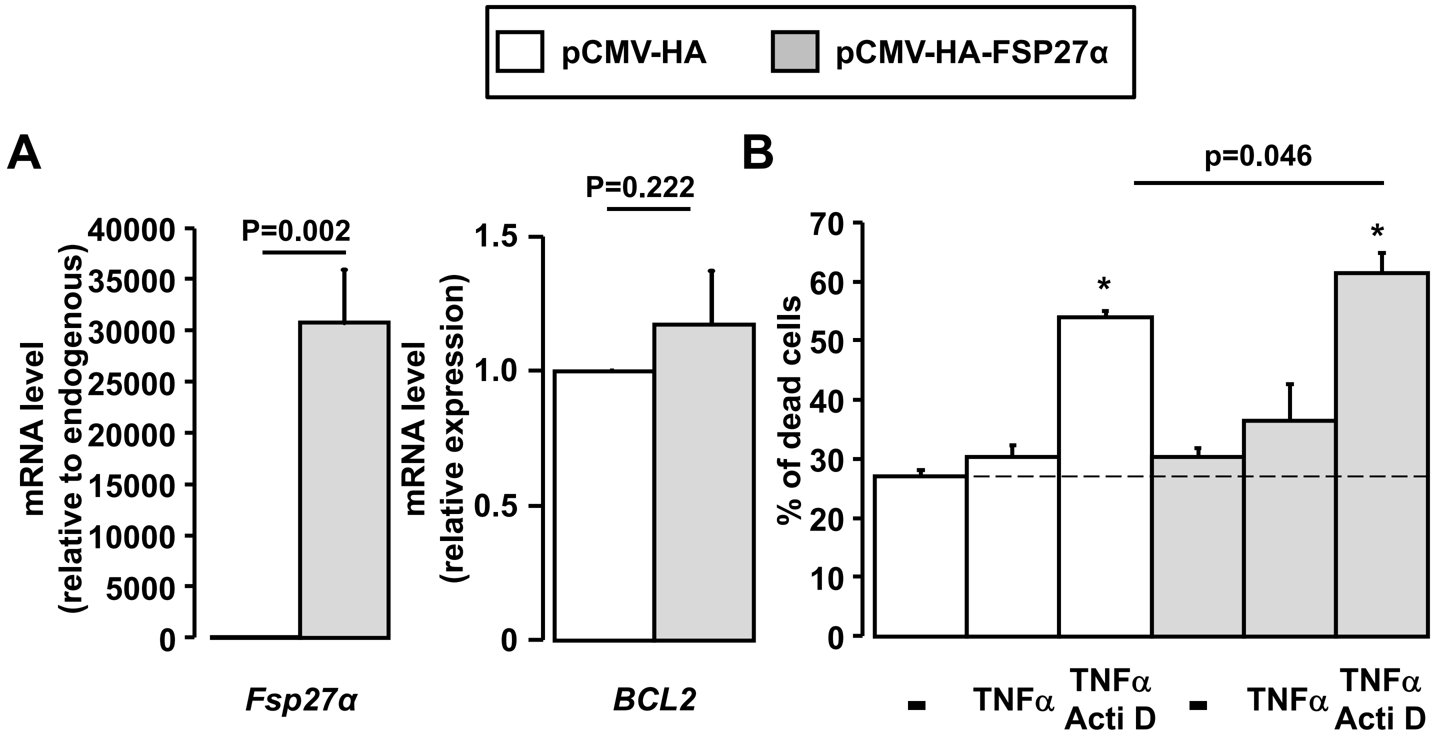

Supplementary Figure 4 : Overexpression of FSP27α enhanced cell death in response to TNFα in HEPG2 hepatocytes. (A) HEPG2 cells were transfected with pCMV-HA and pCMV-HA-FSP27α as indicated. After 48h, the human BCL2, mouse Fsp27 and human CIDEC mRNA expression levels were analyzed by real-time quantitative PCR. The gene expression values were normalized to RPLP0 mRNA levels **(n=3)**. **(B)** After overexpression of FSP27α with pCMV-HA-FSP27α transfection in HEPG2 cells, cell death (flow cytometry)(n=3) were evaluated in the basal state and in response to TNFα (20ng/ml) and actinomycin D (0.1µg/ml) with TNFα (20ng/ml) for 16h. Results relative to the control (pCMV-HA) are expressed as means ± SEM. Data were statistically analyzed using the Student's t-test. *, versus pCMV-HA $p<0.05$.

Unmodified Gels

Figure 1

Anti-HSP90 antibody

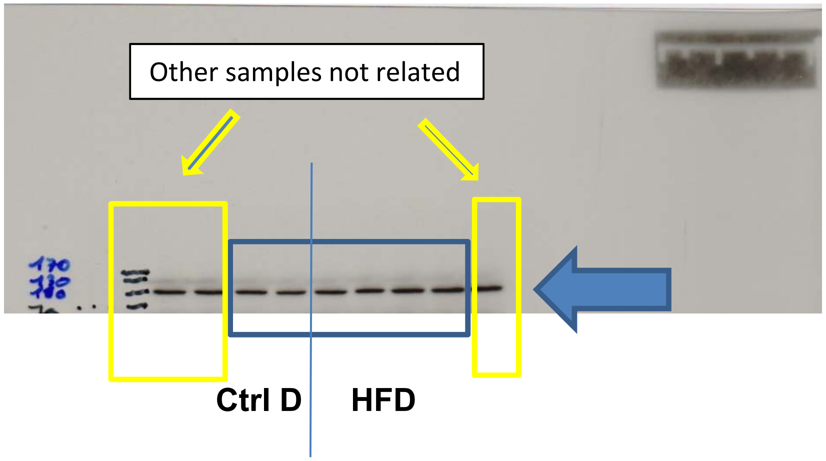

Figure 1

Anti-CIDEA antibody

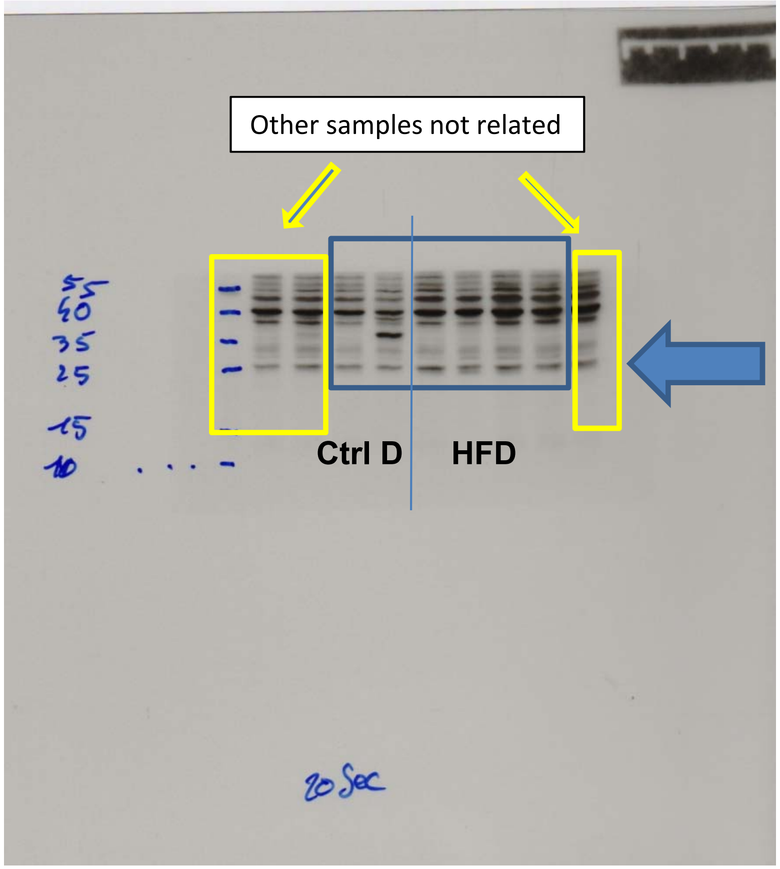

Figure 1

Anti-CIDEC antibody

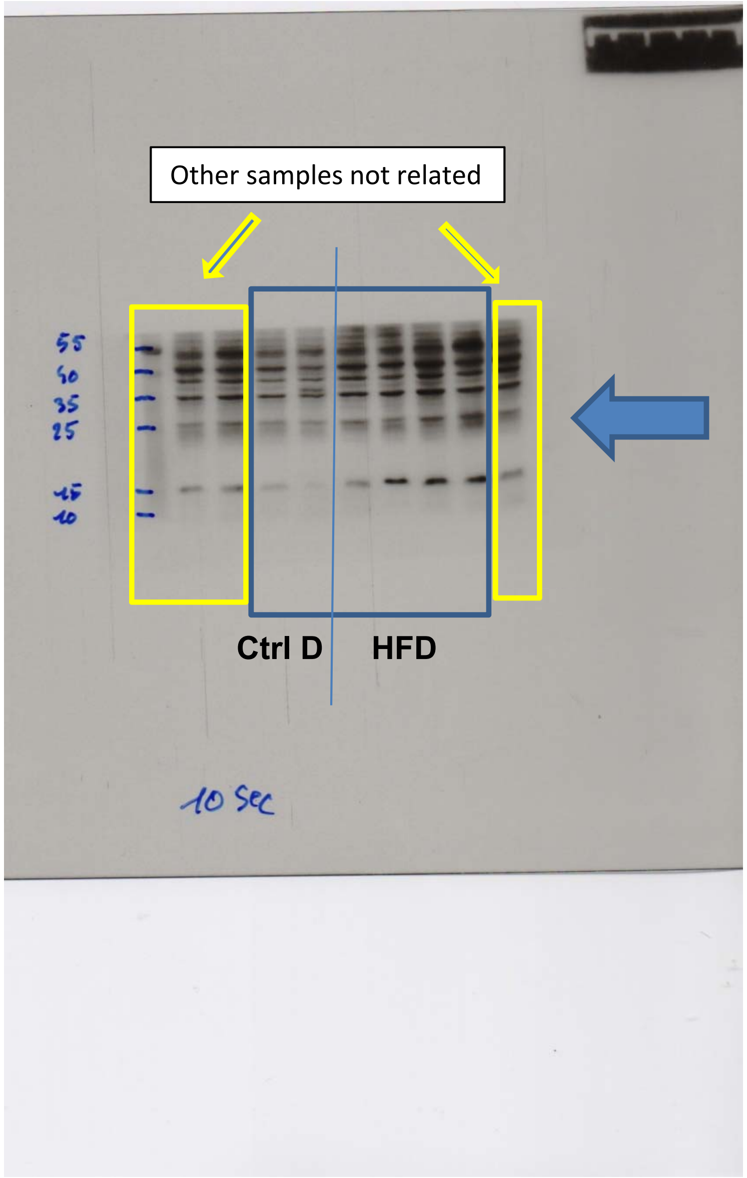

Figure 3

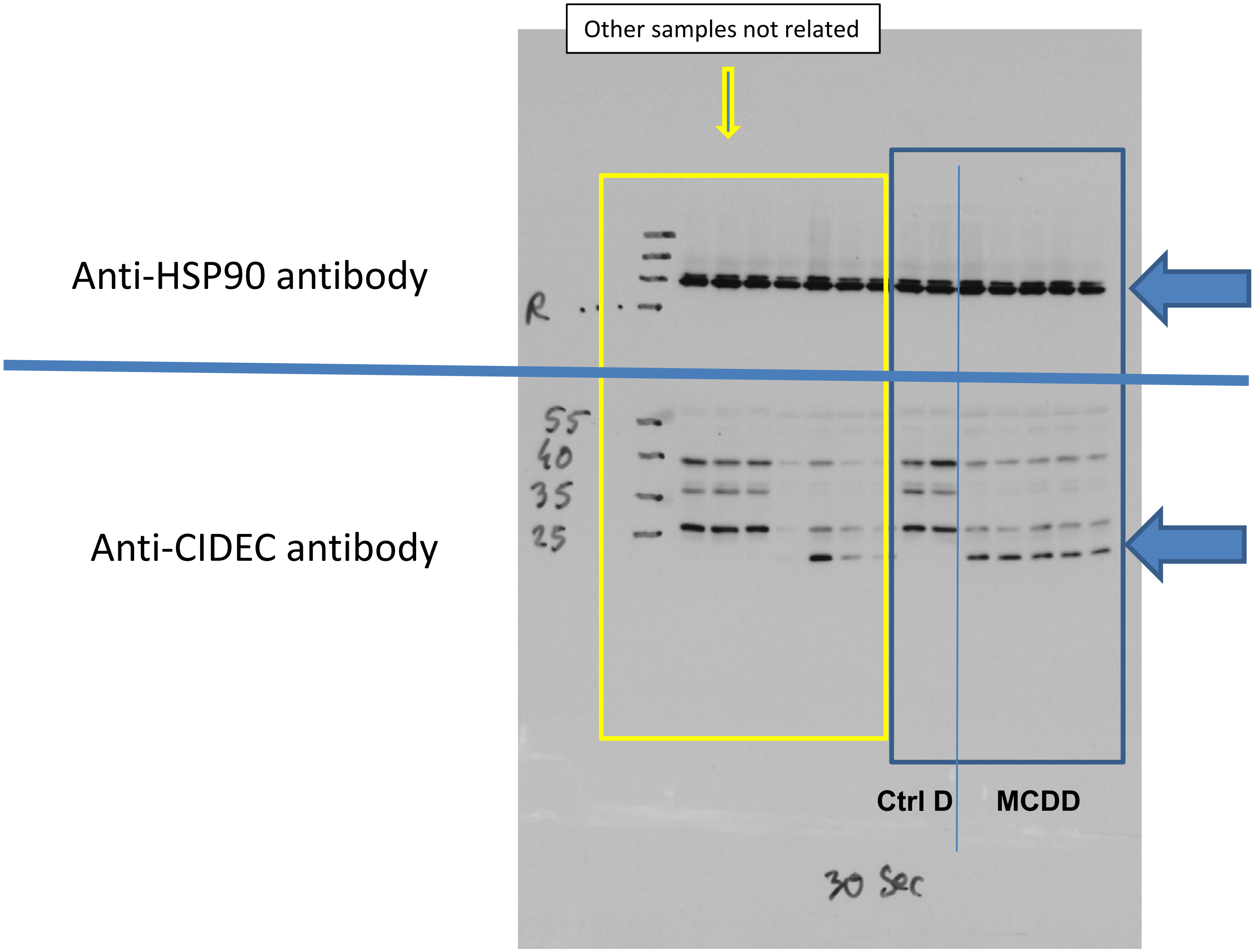
Other samples not related
Anti-HSP90 antibody
R
55
40
35
25
Anti-CIDEC antibody
Ctrl D
MCDD
30 Sec

Figure 3

Anti-CIDEA antibody

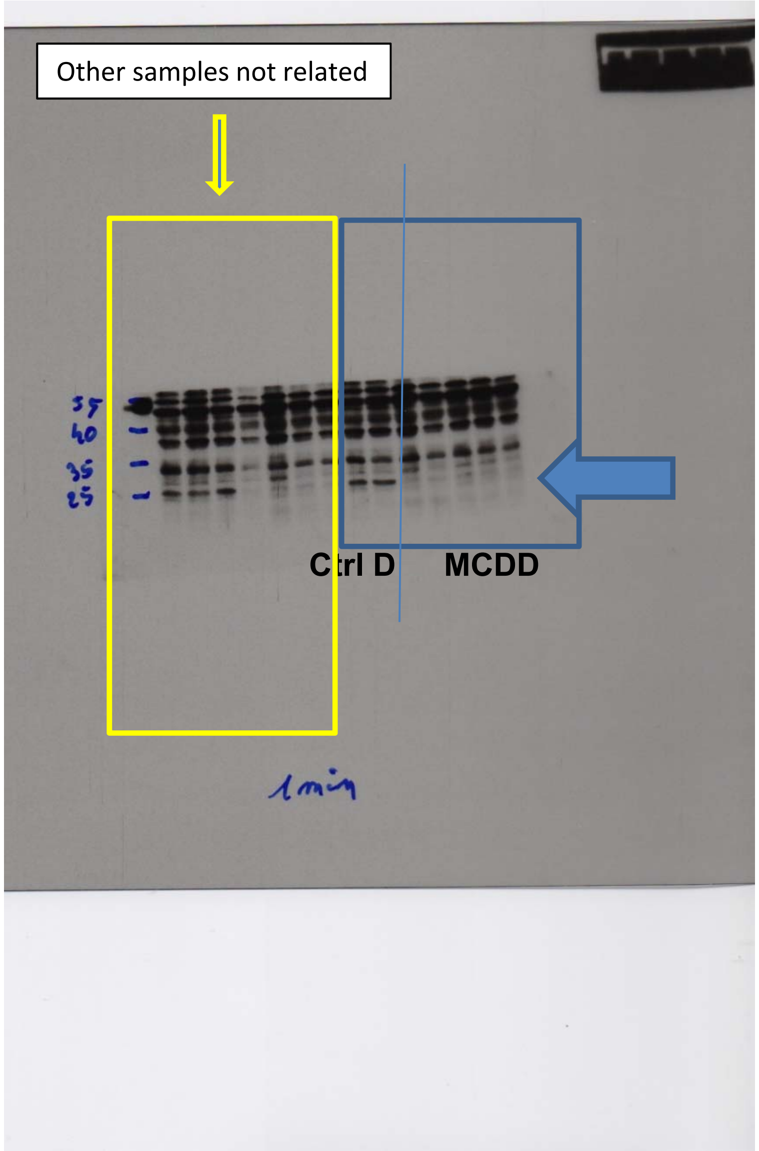

Supplement: Supplementary file 1 — Supplementary data and methods, unmodified gels [file 41598_2019_43928_MOESM1_ESM.pdf]
